# BAFF signaling drives interstitial transformation of mouse renal tubular epithelial cells in a Pin1-dependent manner

**DOI:** 10.1007/s11626-021-00598-y

**Published:** 2021-06-14

**Authors:** Haiyan Xu, Dan Song, Renfang Xu, Xiaozhou He

**Affiliations:** Urology Department, Third Affiliated Hospital of Soochow University, 185# Juqian Street, Changzhou, 213003 China

**Keywords:** BAFF, Renal tubular epithelial cells, Interstitial transformation, Pin1

## Abstract

Aberrant expression of B cell–activating factor belonging to TNF superfamily (BAFF) and its receptors results in abnormal biological activities in hematopoietic and non-hematopoietic cells and is closely associated with the occurrence and development of various diseases. However, the biological significance and potential mechanisms underlying BAFF signaling in renal tubular epithelial cells (RTECs) remain unknown. This study aimed to investigate the biological role of BAFF signaling in RTECs. Mice primary RTECs were applied. The proliferation status and apoptotic rates were examined by MTS assay and flow cytometry, respectively. The expression of BAFF and its receptors was analyzed via flow cytometry and sodium ion transport function, and cytokeratin-18 expression was detected through immunofluorescence staining. In addition, Pin1 was knocked down via siRNA and its expression was assessed through reverse transcription PCR. Lastly, western blotting was performed to analyze E-cadherin, ɑ-SMA, and Pin1 expression. Results suggested that BAFF-R was significantly upregulated upon IFN-γ stimulation, and enhancement of BAFF signaling promoted cell survival and reduced their apoptotic rate, while simultaneously reducing the epithelial phenotype and promoting the interstitial transformation of cells. Furthermore, Pin1 was significantly increased, along with the upregulation of BAFF signaling in the RTECs, and participated in interstitial transformation induced by BAFF signaling. Collectively, the present results elucidate the potential mechanism of loss of normal function of RTECs under long-term high dose of BAFF stimulation provides a potential therapeutic target for renal interstitial fibrosis, and underlining mechanisms of shortening of long-term outcomes of kidney allografts via augmenting of BAFF signaling.

## Background

B cell–activating factor belonging to TNF superfamily (BAFF) is a type II transmembrane protein belonging to the tumor necrosis factor superfamily and is highly conserved across different species. Physiological expression levels of BAFF are generally low in myeloid cells. In the presence of cytokines, such as IFN-γ or TNF-α, BAFF is significantly upregulated (Schneider *et al*. 1999). BAFF has three candidate receptors, BAFF receptor (BAFF-R), transmembrane activator and CAML interactor (TACI), and B cell-maturation antigen (BCMA). Among these, BAFF-R can only specifically bind to BAFF. These receptors are usually expressed on B cells at different stages of development. Different biological phenomena occur when BAFF binds to each of these receptors. The BAFF/BAFF-R signaling pathway transduces an important survival signal to late-transitional B lymphocytes and sustains their self-stabilization (Mackay and Schneider, 2009).

However, BAFF and its receptors are also expressed and play physiological roles in non-hematopoietic cells. For instance, salivary epithelial cells express BAFF when stimulated with dsRNA viruses (Ittah *et al*. 2008; Ittah *et al*. 2009), and the signal is transduced through the BAFF/BAFF-R pathway promotes the survival of these cells in an autocrine manner (Lahiri *et al*. 2014). Human respiratory epithelial cells express BAFF upon activation of Toll-like receptor signaling (McNamara *et al*. 2013; Alturaiki *et al.* 2018), and aberrant BAFF expression is potentially involved in local IgE production (Xu *et al*. 2008). BAFF is upregulated in lesional keratinocytes in individuals with cutaneous lupus erythematosus, and upregulation of BAFF/BAFF-R signaling potentially influences the biological activity of endothelial progenitor cells in systemic lupus erythematosus (SLE) (Jackson and Davidson, 2019). Furthermore, renal tubular epithelial cells (RTECs) express BAFF, which is associated with the histopathological activity of diffuse proliferative lupus nephritis, and in vitro assays have revealed an autocrine loop of BAFF with its cognate receptors on RTECs (Schwarting *et al*. 2018).

During kidney transplantation, BAFF upregulation is closely associated with the rejection of renal transplants (Friebus-Kardash *et al*. 2018). Generally, upregulation of BAFF signaling leads to overactivation and/or prolonged survival of B cells, or T cells, resulting in immunopathological injury (Mackay and Schneider, 2009). We previously reported that BAFF and its receptors are aberrantly upregulated on RTEC in renal allografts, which is closely correlated with the progression of chronic rejection (Xu *et al.* 2012).

Chronic antibody-mediated rejection, a particular type of inflammatory response, is the major cause of graft loss (Lefaucheur and Loupy, 2018). Along with rejected allografts, renal interstitial fibrosis and tubular atrophy (IF/TA) are the two characteristic manifestations. RTECs may not only be victims but also active participants in graft rejection (Nguan and Du, 2009). As a forgoing barrier, RTECs are subjected to various exogenous and endogenous stimuli, which they are susceptible to (Lu *et al*. 2019; Wang *et al*. 2019). This raises the question of whether BAFF signaling plays an important role in the morphology and function of RTECs during renal allograft antibody-mediated rejection. Local renal interstitial inflammation can induce interstitial transformation of RTECs during interstitial fibrosis. However, no studies have investigated whether aberrant upregulation of BAFF signaling in RTECs involves in the epithelial-mesenchymal transition (EMT) of RTECs. Such information would not only facilitate further studies on the mechanism underlying the mesenchymal transition of RTECs, the biological function of BAFF signaling but also elucidate the progression of renal tubular atrophy and renal interstitial fibrosis in renal transplant allografts. Pin1 is one of the molecules regulated by BAFF signaling in malignant B cells (Secreto *et al*. 2014), and it may play key role during EMT induction (Nakada *et al*. 2019). While no direct evidence is available regarding the role of Pin1 on the characteristics of RTECs upon upregulation of BAFF signaling.

In this study, mouse primary renal tubular epithelial cells were cultured, and in vitro analyses were performed to investigate the potential role of BAFF signaling in RTECs, and the function of Pin1 was also explored.

## Materials and methods

### Animals

Six-to-eight-week-old male C57BL/6 mice were purchased from CAVENS (SCXK(SU)2-11-003) and housed in a specific pathogen-free facility under a 12:12-h light-dark cycle with ad libitum access to food and water. Animal experiments were approved by the Academic Committee of Soochow University.

### Culturing of primary RTECs

RTECs were extracted from mice using previously reported methods (Valente *et al*. 2011). In brief, mice were euthanized, and the kidneys were immediately excised and placed in ice-cold complete media in HBSS (Hyclone, Marlborough, MA) supplemented with 1% penicillin-streptomycin (Hyclone). Thereafter, the renal capsule was dissected out, and sagittal sections of the renal cortex were obtained and the medulla was discarded. Further, cortical tissue was minced in complete medium with collagenase I (1 mg/mL, Sigma-Aldrich, St. Louis, MO) and soybean trypsin inhibitor (100 μg/mL, Sigma-Aldrich) and incubated at 37°C for 45 min with frequent mixing. The same volume of ice-cold HBSS was added into tubules to terminate the digestion step. The digestion product was sieved successively with 150-μm and 75-μm mesh sieves, and the filtrate was collected and seeded on collagen-treated plates in a selection culture medium comprising DMEM:F12 culture media (Gibco, Waltham, MA) supplemented with 10 mL/L penicillin-streptomycin (Hyclone), 50 nmol/L hydrocortisone (Sigma-Aldrich), epidermal growth factor, insulin/transferring/selenium (10 μg/mL/5.5 μg/mL, Gibco), and tri-iodothyronine (Gibco), in an incubator (37°C, 5% CO_2_) and cultured to 80% confluence for subculturing. Cells of the second and third passages were used for subsequent experiments.

### Cell proliferation and apoptosis assays

In total, 8×10^4^ cells/well of primary RTECs were seeded in a 96-well plate. The stimulated groups were cultured with rBAFF (2 ng/mL, 5 ng/mL, and 20 ng/mL, respectively, R&D, Minneapolis, MN), or 20 ng/mL rBAFF (R&D) after stimulation with 200 ng/mL BAFF-R-Fc fusion protein (R&D) for 30 min. Cells without cytokines treatment or 200 ng/mL BAFF-R-Fc fusion protein treatment alone were set as controlled groups.

For the cell proliferation assay, after 48 h of incubation, MTS reagents (3-(4,5-dimethylthiazol-2-yl)-5-(3-carboxymethoxyphenyl)-2-(4-sulfophenyl)-2H-tetrazolium, Promega, Madison, WI) were added in each well. After 4 h, OD_490_ values were detected using a SPECTRA Max 384 reader (Spectra Max 384, Molecular Devices, San Jose, CA).

For the cell apoptosis assay, the stimulated cells were cultured with 20 ng/mL rBAFF, and the inhibited groups were cultured with 500 ng/mL BAFF-R-Fc fusion proteins (R&D) for 30 min and then treated with 20 ng/mL rBAFF (R&D). After 48 h, cells were harvested and treated using the protocol of Multiscience (Hangzhou, China). In brief, 2×10^5^ cells/tube of treated primary RTECs was diluted with 500 μL 1× binding buffer, and 5 μL Annexin V-FITC and 10 μL PI were added, gently mixed, and incubated at ambient temperature for 5 min. Thereafter, cells were analyzed through flow cytometry (FCM). Experiments were performed in triplicate.

### Analysis of BAFF and its receptor expression in RTECs by FCM

In total, 8×10^4^ cells/well of primary RTECs were seeded in a 24-well plate and cultured at 37°C in 5% CO_2_ in a conditioned medium. Thereafter, 500 U/mL IFN-γ (R&D) was supplemented in the medium when approached 80% confluence. After 48 h, cells were digested with 0.25% trypsin and centrifuged for 5 min at 1000 r/min (the centrifugation radius was 8cm). The cell pellets were resuspended in PBS containing 5% FBS, and BAFF and BAFF-R expression levels were assessed through FCM.

Approximately 2×10^5^ cells were incubated with FITC-labeled rat-anti-mBAFF antibody (Abcam, Cambridge, MA) or PE-labeled anti-mBAFF-R (BD, Franklin Lakes, NJ), anti-mBCMA (R&D), and anti-mTACI (Abcam) antibody in each well for 30 min at 4°C. Control cells were cultured in medium not containing IFN-γ. Experiments were performed in triplicate.

### Cytokeratin (CK)-18 detection

To determine the expression level of cytokeratin-18 (CK-18) in RTECs, the cells were serum-starved overnight in DMEM:F12, or treated with 500 ng/mL BAFF-R-Fc fusion protein (R&D) for 30 min and 5 ng/mL recombinant BAFF (rBAFF, R&D) or stimulated with 5 ng/mL rBAFF alone. Cells without cytokines treatment or 500 ng/mL BAFF-R-Fc fusion protein treatment alone were set as controlled groups. When cells were cultured to 90% confluence (approximately 48 h), they were stained with rabbit anti-mCK-18 polyclonal antibody (Sigma-Aldrich). In brief, the following method was used: treatment with 4% paraformaldehyde for 15 min; treatment with 0.3% Triton-X-100 at ambient temperature for 10 min; blocking with BSA-TBS at ambient temperature for Sigma-Aldrich at 4°C overnight; four PBS washes, 5 min each; incubation with goat anti-mouse FITC-Ig antibody (1:100, Sigma-Aldrich) for 45 min in the dark; addition of the anti-quencher reagent (Invitrogen, Franklin Lakes, CA); and observing using a fluorescence microscope (Leica, Wetzlar, Germany). Between each procedure, at least three PBS washes were carried out. Fluorescence intensity was analyzed in the images, using ImageJ software (National Institutes of Health, Bethesda, MD). Experiments were performed in triplicate.

### Fluorescein sodium transport test

The reagent dose and experimental groups were the same as that for CK-18 detection experiments. Primary RTECs were cultured to 90% confluence, washed with HBSS (warm-up in a water bath at 37°C) once, and incubated in HBSS at 37°C for 10 min. Thereafter, the cells were incubated with 1 mL HBSS with 1×10^−5^ mol/L fluorescein sodium at 37°C for 20 min. Ice-cold PBS was then added into the wells to terminate the reaction, followed by four rapid washes with ice-cold PBS. Fluorescein staining intensity was determined from fluorescence microscopic (Leica) images through ImageJ. Experiments were performed in triplicate.

### Pin1 siRNA co-transfection

Briefly, primary RTECs were cultured in DMEM:F12 medium without penicillin-streptomycin, and allowed to approach 60–80% confluence. Thereafter, the cells were incubated with Pin1 siRNA in accordance with the manufacturer’s instructions (RiboBio, Guangzhou, China). The final concentration of Pin1 siRNA mixture was 40 nM, of which 400 μL of the final mixture was gently added into each well, and the cells were incubated for 6 h at 37°C in a CO_2_ incubator. Thereafter, 500 μL of 2× completed medium without antibiotics were added in each well, mixed gently, and further incubated for 24 h. Experiments were performed in triplicate.

### Reverse transcription PCR (RT-PCR) analysis

The effects of siRNA were analyzed through RT-PCR. Total RNA was extracted using the RNAiso Plus kit (TaKaRa, Dalian, China) in accordance with the manufacturer’s instructions. cDNA was prepared using PrimeScript 1st Strand cDNA synthesis Kit (TaKaRa). mRNA levels were determined through PCR using Premix Taq (TaKaRa Taq Version 2.0 plus dye, TaKaRa). Electrophoresis was performed to observe the size of the PCR products.

### Western blots

In total, 4×10^5^ cells/well of primary RTECs were seeded in a 96-well plate. In the rBAFF stimulation group, the final rBAFF concentration was 200 ng/mL. In the BAFF-R-Fc and rBAFF sequential stimulation group, cells were first stimulated with 1000 ng/mL BAFF-R-Fc fusion protein for 30 min at 37°C, and then 200 ng/mL of rBAFF was added in the medium. In the Pin1 siRNA-transfected cells, the final concentration of Pin1 siRNA was 40 nM. In the Pin1 siRNA-transfected and rBAFF-stimulated cells, after primary RTECs were transfected with 40 nM Pin1 siRNA for 24 h, the medium was discarded and replaced by a completed medium containing 200 ng/mL rBAFF. In Pin1 siRNA-transfected, BAFF-R-Fc-treated, and rBAFF-stimulated cells, the same aforementioned procedure was followed, with Pin1 siRNA being substituted with control siRNA or no siRNA among the control cells.

After 48 h of culture under final conditions, the total cell lysates were isolated using RIPA buffer (Sigma-Aldrich) with a protease and phosphatase inhibitor cocktail (Thermo Scientific, Franklin Lakes, CA), and quantified using the Enhanced BCA Protein Assay kit (Beyotime, Nantong, China).

Proteins were through SDS-PAGE and electro-transferred onto a nitrocellulose membrane (Bio-Rad, Hercules, CA). After 1 h of incubation with 5% BSA, the membranes were probed overnight with the following primary antibodies: anti-Pin1 (1:400, Santa Cruz, TX), anti-E-cadherin (1:1000, CST, Danvers, MA), anti-α-SMA (1:1000, CST), and anti-β-actin (1:1000, CST) antibodies. After washing, the membranes were probed with an HRP-conjugated secondary antibody (1:1000, CST) in blocking solution for 1 h at ambient temperature. HRP activity was assessed using the chemiluminescence kit (Pierce, Appleton, WI) in accordance with the manufacturer’s instructions. The images were analyzed using Image Lab Software (Bio-Rad).

### Statistical analysis

Data are presented as mean (x̄) ± SD values and analyzed using SPSS 19.0 software (SPSS Inc., Chicago, IL). Between-group comparisons were performed using a paired samples *t* test, with *P*<0.05 considered statistically significant.

## Results

### Identification of primary mouse RTECs

**Figure 1.**
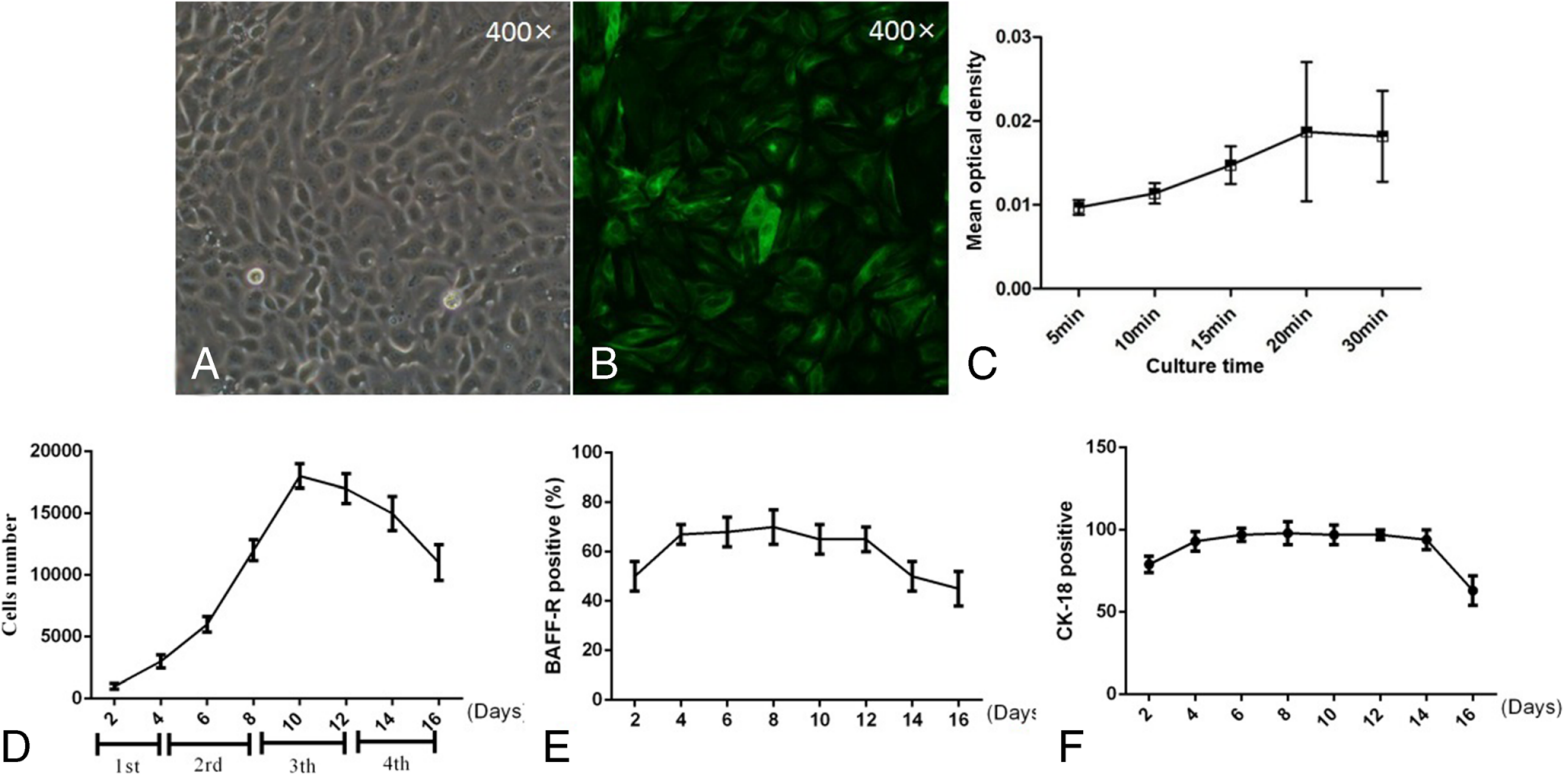
Identification of mice primary renal tubular epithelial cells (RTECs). (*A*) The cells displayed a typical polygonal cobblestone-like morphology, with good refraction and a relatively large cell size (400×). (*B*) Immunofluorescence staining revealed that the rate of CK-18 positivity in the primary cells was >90%. (*C*) The ability of Na^+^ transfer was also tested through an immunofluorescence assay. Intracellular fluorescence intensity gradually increased with time. (*D*) The growth curve of primary RTECs. The cells took about 4 d to overgrow the flask and continued to grow well for nearly 10 d. (*E*) BAFF-R expression was detected by flow cytometry every 2 d. BAFF-R was stably expressed on primary RTECs during the initial 14 incubation days, and then it went down. (*F*) CK-18-positive population (%) through the initial 16 incubation days, stained by immunofluorescence assays. It was shown that CK-18 was stably expressed for about 14 d, and then it went down.

The primary cells we obtained had a typical polygonal cobblestone-like morphology, with tight junctions among them, with good refraction and relatively large sizes (Fig. 1*A*).

CK-18 was particularly expressed on epithelial cells, potentially serving as a biomarker of primary RTECs. Immunofluorescence staining revealed that the rate of CK-18 positivity in the primary cells was >90% (Fig. 1*B*). Ion transport function is another important function of epithelial cells. We investigated the Na^+^ transferability through immunofluorescence. We observed a gradual increase in the intracellular fluorescence intensity with time (Fig. 1*C*).

To observe the growth status of primary RTECs, we counted the cell number every 2 d. The results suggested that cells could grow well for about 14 d (3 generations) (Fig. 1*D*). And BAFF-R expression was stably on primary RTECs through the initial 3 generations, and then it went down (Fig. 1*E*). Moreover, the expression rate of CK-18 was basically consistent with BAFF-R (Fig. 1*F*).

### BAFF-R was significantly upregulated on primary RTECs after IFN-γ stimulation

**Figure 2.**
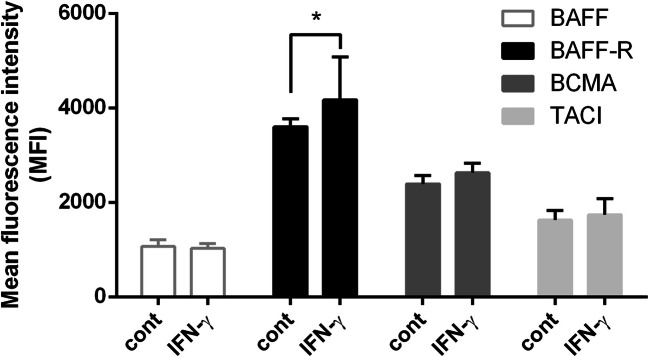
BAFF-R was significantly upregulated on primary renal tubular epithelial cells (RTECs) after IFN-γ stimulation. Flow cytometry assays indicated that the mean fluorescence intensity of BAFF-R on RTECs significantly increased after IFN-γ stimulation (^*^P<0.05). However, the expression of BAFF, BCMA, and TACI expression did not vary significantly (*P*>0.05).

FCM assays revealed a significant increase in the mean fluorescence intensity (MFI) of BAFF-R on RTECs after IFN-γ stimulation (4166.25±913.91 vs 3602.75±162.47.58, respectively; *P*<0.05); however, the expression of BAFF, BCMA, and TACI did not vary significantly (1027.40±98.5 vs 1066.40±143.42, 2392.32±178.21 vs 2543.23±209.18, 1629.84±197.35 vs 1737.64±338.05, respectively; *P*>0.05) (Fig. 2).

### BAFF upregulation promoted proliferation and inhibited apoptosis in primary RTECs

**Figure 3.**
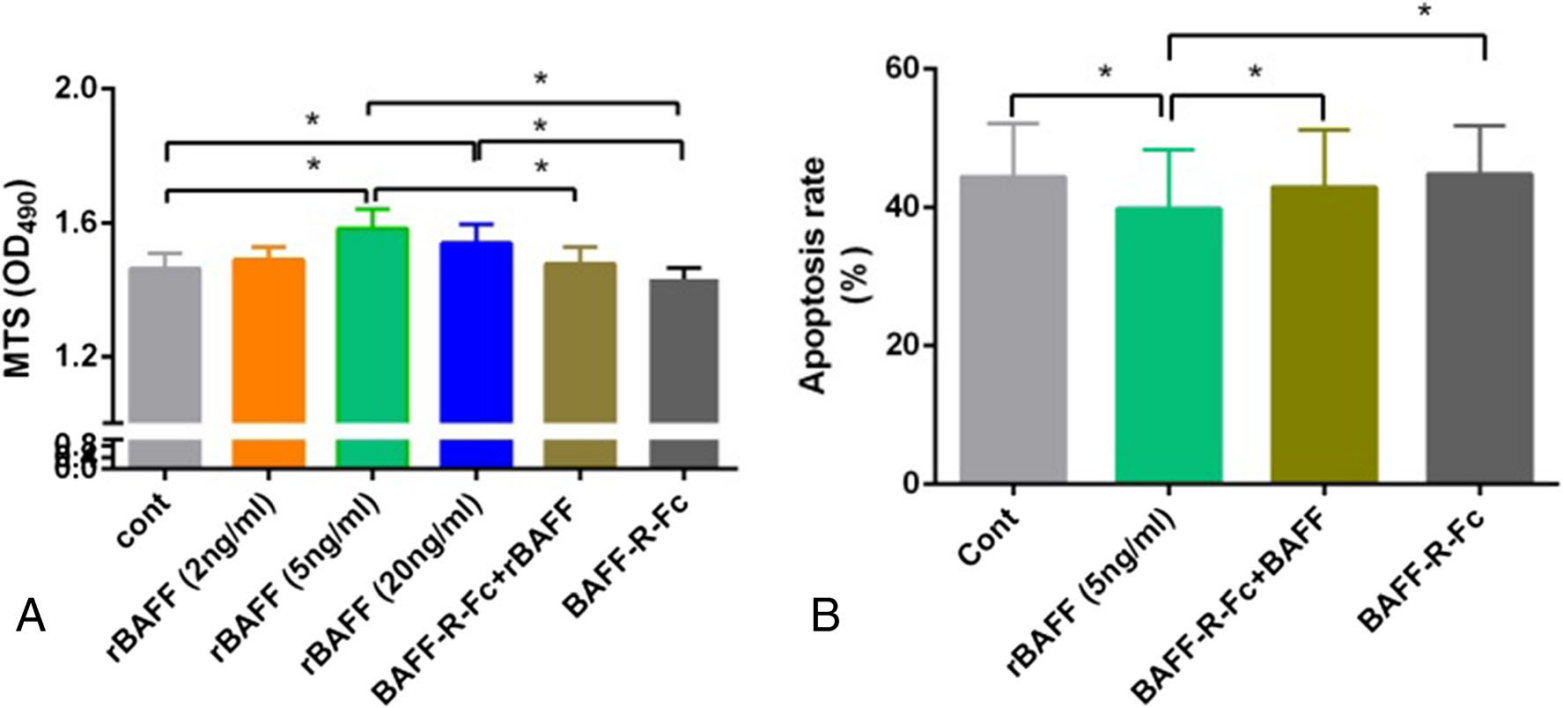
Upregulation of BAFF signaling promotes proliferation and decreases apoptosis in primary renal tubular epithelial cells (RTECs). (*A*) MTS assay indicated that 5 ng/mL and 20 ng/mL rBAFF significantly promoted proliferation in primary RTECs (^*^P<0.05, respectively), but the effect was not dose-dependent; pre-treatment with BAFF-R-Fc fusion protein significantly mitigated the effect of rBAFF on RTECs, compared to 5 ng/mL rBAFF treatment (^*^P<0.05). (*B*) rBAFF stimulation significantly inhibited apoptosis in RTECs, compared to the control groups (^*^P<0.05). Furthermore, pre-treatment with BAFF-R-Fc fusion protein significantly reduced the effect of rBAFF on RTECs (^*^P<0.05).

The MTS assay revealed that, compared with blank control group (cont) and BAFF-R-Fc alone treatment group, 5 ng/mL and 20 ng/mL rBAFF significantly promote proliferation in primary RTECs (*P*<0.05), but the effect was not dose-dependent. Pre-treatment with BAFF-R-Fc fusion protein significantly mitigated the effect of rBAFF on RTECs, compared to treatment with 5 ng/mL rBAFF (1.477±0.050 vs 1.532±0.058, respectively; *P*<0.05) (Fig. 3*A*)

However, rBAFF stimulation significantly inhibited apoptosis in RTECs compared to control groups and BAFF-R-Fc alone treated groups (39.850%±8.544% vs 44.467%±7.642%; 39.850%±8.544% vs 44.86±7.02, respectively, *P*<0.05). Furthermore, pre-treatment with BAFF-R-Fc fusion protein significantly mitigated the pro-apoptosis effect of rBAFF on RTECs (42.950%±8.330% vs 39.85%±8.544%; *P*<0.05) (Fig. 3*B*).

### Upregulation of BAFF signaling reduced the epithelial characteristics of primary RTECs

**Figure 4.**
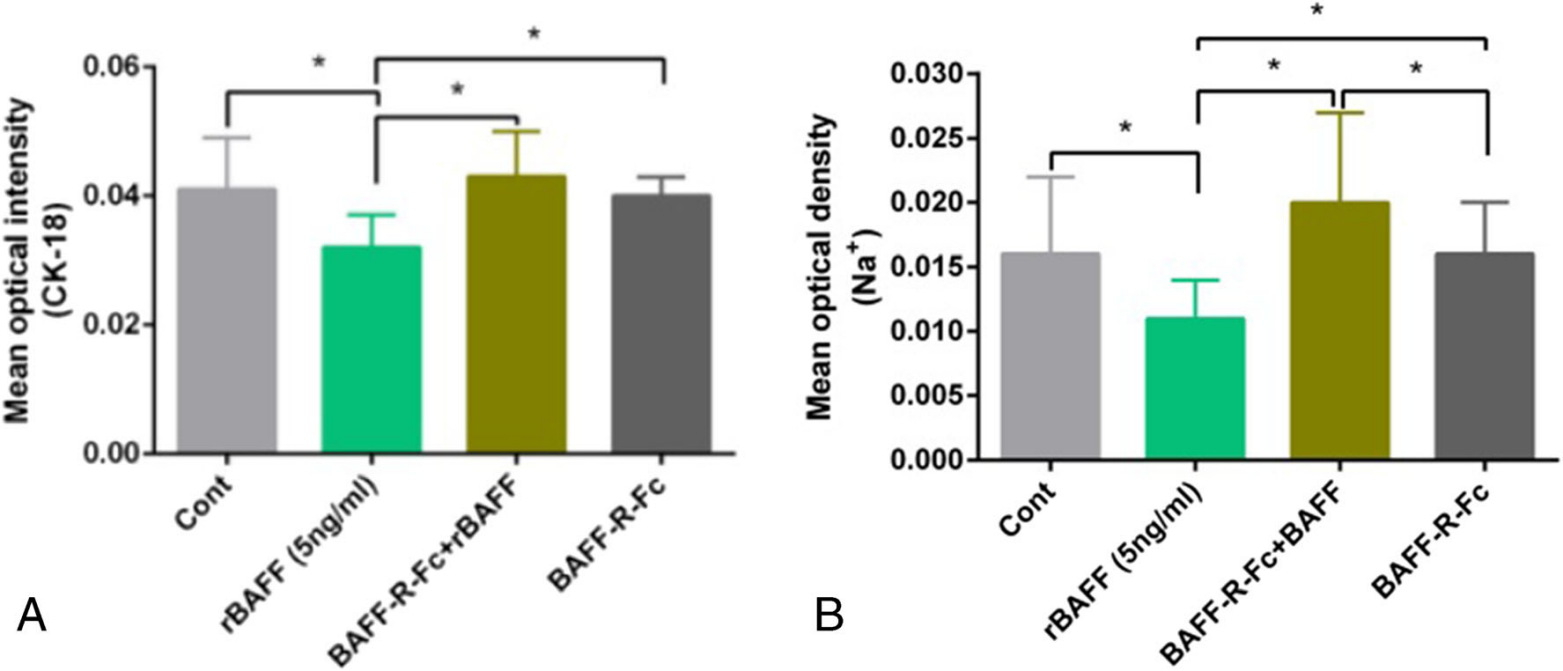
Upregulation of BAFF signaling decreased the epithelial characteristics of primary renal tubular epithelial cells (RTECs). (*A*) BAFF upregulation reduced CK-18 expression, compared to the control cells (^*^P<0.05); however, pre-treatment with BAFF-R-Fc fusion protein significantly mitigated the effect of BAFF on CK-18 expression, compared to rBAFF treatment (^*^P<0.05). (*B*) BAFF upregulation significantly decreased the transferability of Na^+^ in RTECs (^*^P<0.05). Furthermore, pre-treatment with BAFF-R-Fc fusion protein significantly rescued Na^+^ transferability in RTECs, which had initially decreased because of rBAFF stimulation (^*^P<0.05).

While BAFF upregulation reduced CK-18 expression, compared to the control cells and BAFF-R-Fc fusion protein–treated cells (0.032±0.005 vs 0.041±0.008; 0.032±0.005 vs 0.04±0.003. respectively; *P*<0.05), pre-treatment with BAFF-R-Fc fusion protein significantly mitigated the effect of BAFF on CK-18 expression, compared to rBAFF treatment (0.043±0.007 vs 0.032±0.005, respectively; *P*<0.05) (Fig. 4*A*).

Furthermore, BAFF upregulation significantly decreased Na^+^ transferability in RTECs, compared to the control group and BAFF-R-Fc fusion protein treatment group (0.011±0.003 vs 0.016±0.006; 0.011±0.003 vs 0.016±0.004, respectively; *P*<0.05). Moreover, pre-treatment with BAFF-R-Fc fusion protein significantly rescued Na^+^ transfer activity in RTECs, which had initially decreased because of rBAFF stimulation (0.020±0.007 vs 0.011±0.003, respectively; *P*<0.05) (Fig. 4*B*).

### Upregulation of BAFF signaling induced a mesenchymal transition in a Pin-1-dependent manner

**Figure 5.**
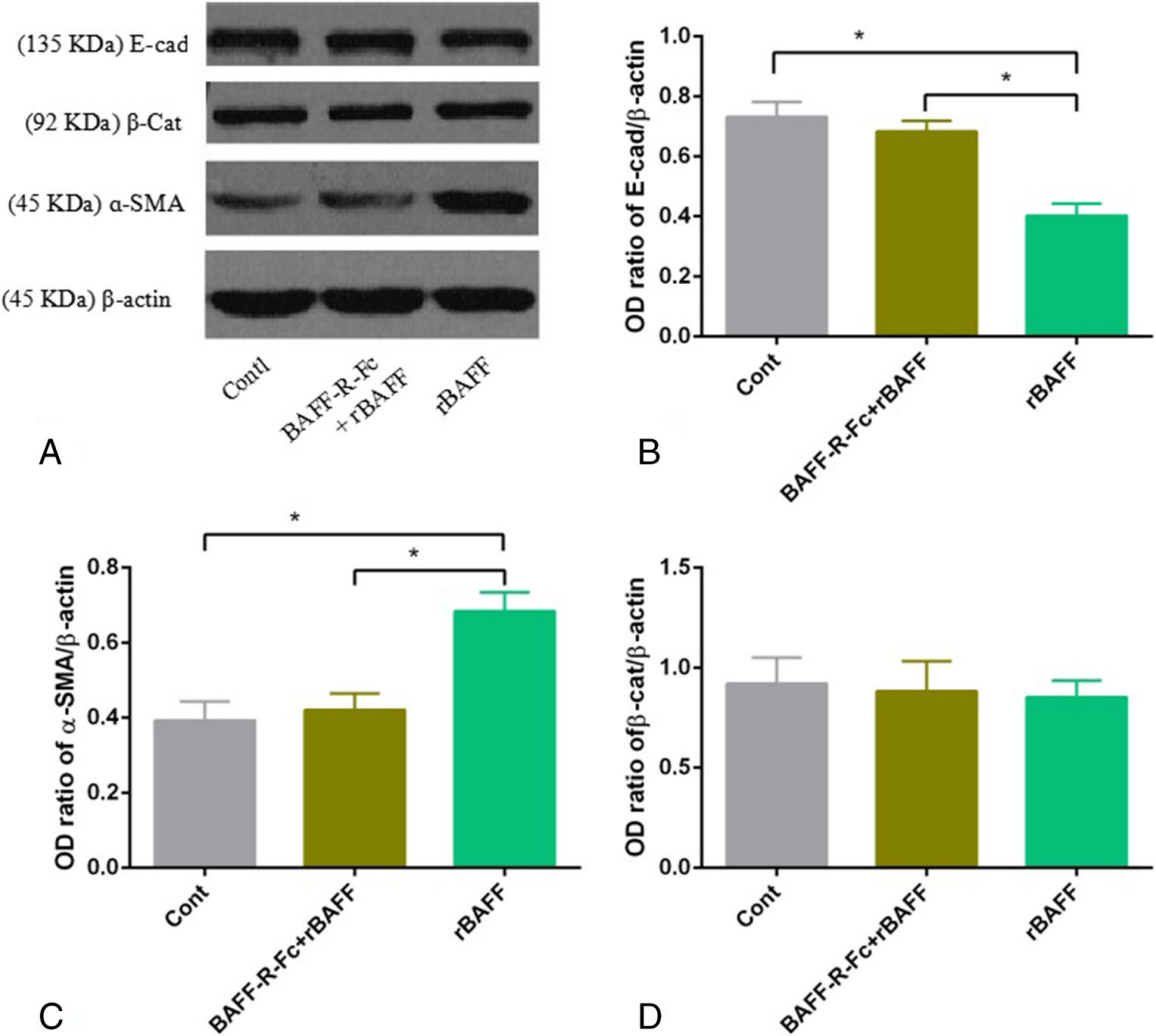
Upregulation of BAFF signaling stimulated interstitial transformation in primary renal tubular epithelial cells (RTECs). (*A*) The original western blot. The experimental groups were rBAFF stimulation, BAFF-R-Fc protein treatment and rBAFF stimulation group, and control group. E-cadherin (E-cad), β-catenin (β-cat), and ɑ-SMA were detected, and β-actin was considered the internal reference. (*B*, *C*, *D*) The results of grayscale scanning indicated that rBAFF significantly downregulated E-cadherin but significantly upregulated ɑ-SMA, and pre-treatment of BAFF-R-Fc protein mitigated the effect of rBAFF on primary RTECs (^*^P<0.05). No significant effect of rBAFF and/or BAFF-R-Fc protein treatment was observed.

E-cadherin and ɑ-SMA expression were assessed to analyze the effect of the upregulation of BAFF/BAFF-R signaling on RTECs. In this study, stimulation with a high concentration of rBAFF significantly downregulated E-cadherin (0.403±0.04 vs 0.744±0.05, *P*<0.05), but upregulated α-SMA in the primary RTECs (0.684±0.043 vs 0.393±0.051, *P*<0.05). Furthermore, the effect of rBAFF could be mitigated by BAFF-R-Fc fusion protein (0.684±0.035 vs 0.733±0.05 and 0.421±0.043 vs 0.393±0.051, respectively; *P*<0.05) (Fig. 5).

Pin1 retained the WW domains (residues 1–39) at the N-terminal and the PPIase domain (residues 45–163) at the C-terminals (Verdecia *et al.* 2000; Chen *et al*. 2018). Pin1 reportedly affects substrate stability, and regulates TGF-β1 expression, thus enhancing TGF-β1-mediated fibrogenesis signaling (Matsuura *et al*. 2010; Inoue *et al.* 2019). In cells expressing BAFF-R_H159Y_, rBAFF stimulation increased the growth and survival of cells through the regulation of relevant genes including Pin1 (Secreto *et al*. 2014).

**Figure 6.**
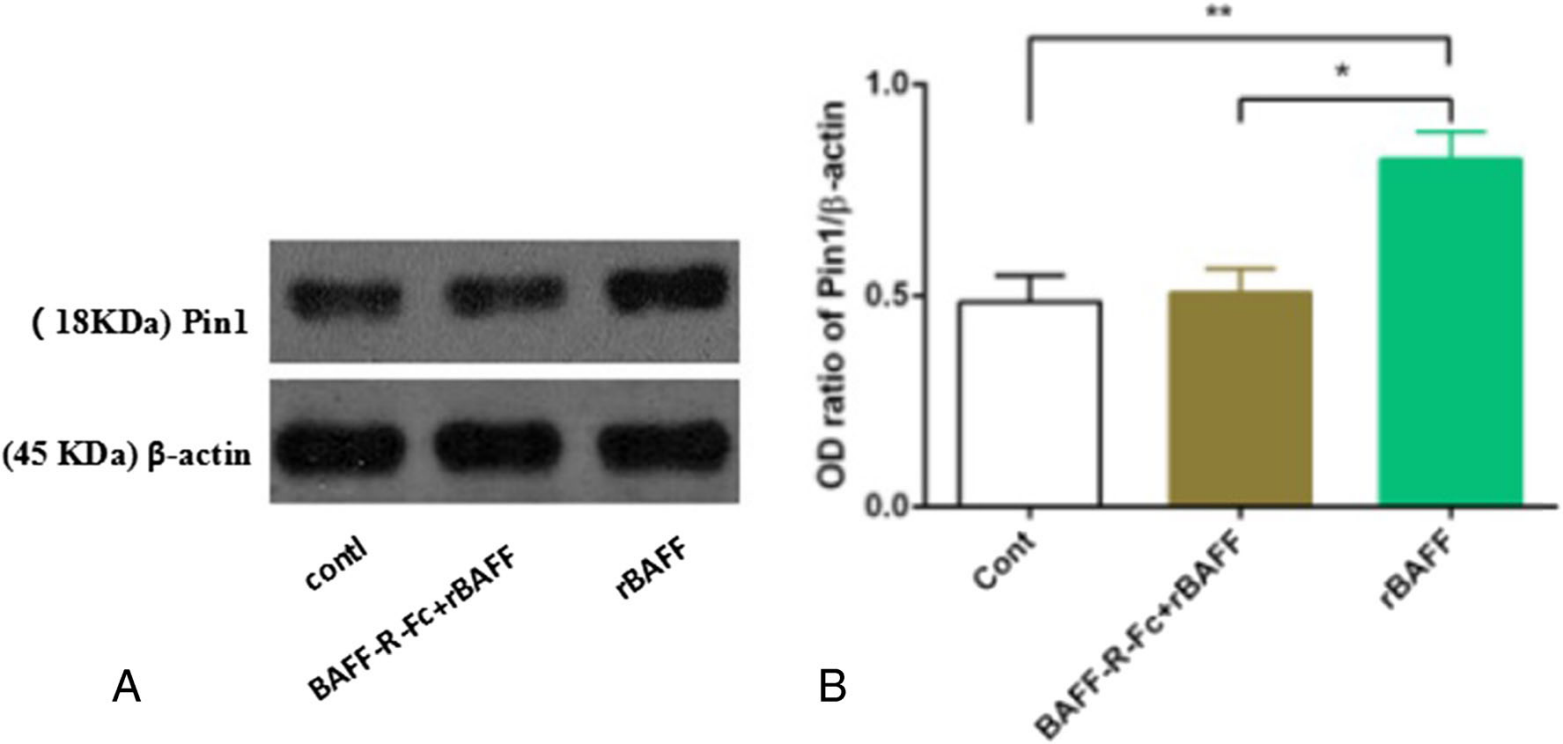
Pin1 was upregulated with the upregulation of BAFF signaling. (*A*) The original images of western blot. The experimental groups were the same as those indicated in Fig. 5. (*B*) The grayscale scanning results indicate that rBAFF stimulation significantly upregulated the expression of Pin1 (^*^P<0.05; ^**^P<0.01).

**Figure 7.**
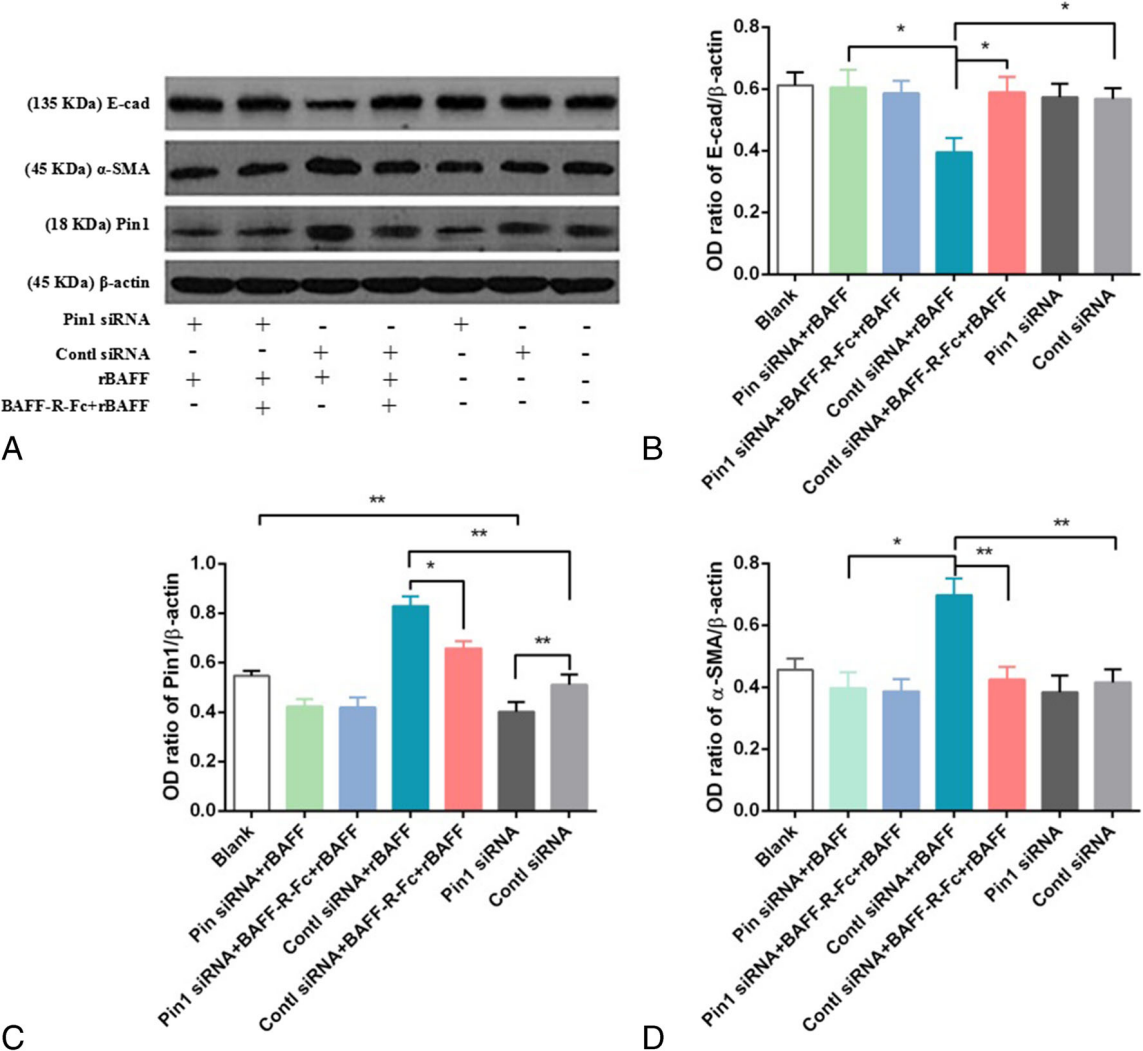
Pin1 is involved in interstitial transformation during BAFF signaling. (*A*) The original images of western blot. The effect of Pin1 siRNA and the control siRNA treatment were observed, in combination with rBAFF and BAFF-R-Fc protein treatment groups. (*B*) The grayscale scanning results suggest that Pin1 siRNA knocked down Pin1 mRNA (^**^P<0.01), and no similar effect of control siRNA was observed (^*^P<0.05; ^**^P<0.01). (*C*) Pin1 siRNA treatment reversed the effect of rBAFF stimulation of downregulating E-cadherin (^*^P<0.05). (*D*) Pin1 siRNA treatment reversed the effect of rBAFF stimulation of upregulating ɑ-SMA (^*^P<0.05; ^**^P<0.01).

Herein, BAFF upregulation in primary RTECs significantly increases Pin1 expression (0.822±0.066 vs 0.486±0.062, respectively; *P*<0.01), and this effect could be inhibited through pre-treatment with BAFF-R-Fc fusion protein (0.508±0.056 vs 0.486±0.062, respectively; *P*<0.05) (Fig. 6). Pin1 siRNA was used to knock down Pin1. The optimal working concentration of Pin1 siRNA was 40 nM (data not shown). In subsequent experiments, Pin1 silencing significantly upregulated E-cadherin and significantly downregulated α-SMA through BAFF stimulation (*P*<0.05). Furthermore, upon pre-treatment with BAFF-R-Fc fusion protein, no similar effects of Pin1 siRNA on RTECs were observed upon BAFF stimulation (*P*< 0.05, Fig. 7).

## Discussion

Renal tubules are a major component of the kidney and are vulnerable to various injuries including ischemia, proteinuria, toxins, and metabolic disorders. In response to injury, RTECs can synthesize and secrete various bioactive molecules that drive interstitial inflammation and fibrosis. Furthermore, RTECs play an active role in progressive renal injury through mechanisms associated with the conversion into collagen-producing fibroblast phenotype, cell cycle arrest at both G1/S and G2/M checkpoints, and metabolic disorder (Allison. 2015; Liu *et al*. 2019). The suppression of interstitial transformation of RTECs can significantly reduce the accumulation of interstitial fibroblasts and mitigate tubule injury, thus delaying the occurrence of renal fibrosis and protecting renal function (Grande *et al*. 2015; Lovisa *et al*. 2015).

In renal allograft fibrosis, tubular atrophy is considered an interstitial fibrosis/tubular atrophy (IF/TA). IF/TA occurs in approximately 40% of kidney allografts at 3–6 mo after transplantation, its incidence increasing to 65% at 2 yr after transplantation (Boor and Floege. 2015). To further enhance the long-term outcome of underlying recipients and allografts, it is necessary to illuminate the pathomechanism. Detailed studies on the mechanism underlying interstitial transformation of RTECs may yield a potential target for clinical treatment of renal interstitial fibrosis.

During interstitial transition in RTECs, epithelial markers including CK-18 and E-cadherin are downregulated or completely obliterated; however, mesenchymal markers including ɑ-SMA are upregulated. This occurs along with a change in cell polarity and the formation of interconnecting intercellular structures and is finally accompanied by decreased cell function (Majo *et al*. 2019).

In this present study, BAFF-R was the only receptor with significantly upregulated expression when RTECs were stimulated by IFN-γ, and the expression of other two receptors, BCMA and TACI, did not vary significantly. When RTECs were challenged by enhancement of BAFF signaling, the ion transfer activity and the epithelial characteristics decreased, but mesenchymal characteristics increased. BAFF can bind to other two receptors, BCMA and TACI, in addition to its specific receptor, BAFF-R. To determine the function of BAFF signal, BAFF-R-Fc fusion protein was applied, which can bind to BAFF specifically and with high affinity. The results suggested that BAFF-R-Fc fusion protein could block or neutralize these effects.

For the rigor of the statement, we considered that BAFF signaling can drive interstitial transformation of mouse renal tubular epithelial cells. The present data obtained from the mouse primary RTECs and the characteristics of cells emulate those under in vivo conditions. Therefore, we considered that BAFF signal enhancement actively promotes interstitial transformation in RTECs. Persistent and aberrant upregulation of BAFF in recipients may be one of the primary causes of kidney allograft failure. Our results potentially provide an experimental basis for BAFF-targeted biotherapy because therapeutics targeting BAFF are now clinically available (Panzer. 2020).

To investigate the potential downstream signaling cascade of interstitial transition after BAFF stimulation, Pin1 was analyzed in RTECs. Pin1 is a unique enzyme, which isomerizes the cis-trans conformation between pSer/pThr and proline residues, thus regulating the function, stability, and/or subcellular distribution of its target proteins, subsequently affecting the function of substrate proteins and participating in the regulation of various cell functions (Nakatsu *et al*. 2016; Nakatsu *et al*. 2020), and the catalytic activity of Pin1 would potentially alter the phosphorylation status of various enzymes (Liu *et al*. 2016).

Pin1 is reportedly involved in the regulation of the epithelial-mesenchymal transition (Kim *et al.* 2009) and the expression of immune-related genes, inflammatory cytokines, and fibrogenic growth factors (Shen *et al*. 2005; Shen *et al*. 2012). Pin1 upregulation in mouse and human fibrotic liver tissues, and Pin1-dependent epithelial-mesenchymal transformation activation pathways are potentially associated with liver fibrosis (Yang *et al*. 2014). Moreover, Pin1 is reported to be a key regulator of extracellular matrix gene expression on administering a high-phosphate diet to mice (Shen *et al*. 2016). The present data indicate that the significant upregulation of Pin1 during interstitial transition of RTECs was induced by enhancement of the BAFF signal, and inhibition of Pin1 significantly weakened the interstitial transformation of RTECs by BAFF signals. These results indicate that Pin1 plays an essential role during interstitial transformation in RTECs induced by BAFF signals.

A novel reciprocal loop of TGF-β1/PML SUMOylation/Pin1 leading to myocardial fibrosis may exist (Wu *et al*. 2019). Inhibition of SUMO-1 by ginkgoic acid alleviated myocardial infarction-induced heart dysfunction and fibrosis, and the SUMOylated PML/Pin1/TGF-β1 pathway is crucial for ginkgoic acid-inhibited cardiac fibrosis, wherein Pin1 functions as a positive regulator of TGF-β1 mRNA (Qiu *et al*. 2018). Because the ultimate goal of our study was to illuminate the relationship between BAFF signal and interstitial degeneration of renal tubular epithelial cells, we did not delve into the downstream signal pathway. This was the weakness of this study. We will continue to study BAFF signaling and transplantation in depth.

## Conclusion

Although the involvement of BAFF signal in renal disease and allograft rejection has been reported (Xu *et al*. 2009; Zheng *et al*. 2017; Kühne *et al*. 2017), our data firstly provide direct evidence that BAFF signal is associated with renal fibrosis. In spite of the present results obtained from a non-transplant background, they not only elucidate the mechanism underlying the role of BAFF expression in the reduction of the long-term outcome of kidney allografts, but also provide a potential therapeutic target for renal interstitial fibrosis.

## Abbreviations

RTECs: renal tubular epithelial cells
BAFF: B cell activating factor belonging to TNF superfamily
BAFF-R: BAFF receptor
TACI: transmembrane activator and CAML interactor
BCMA: B cell-maturation antigen
